# A borderline form of empty follicle syndrome treated with a double-trigger of gonadotropin-releasing hormone agonist and human chorionic gonadotropin: A case report

**DOI:** 10.1097/MD.0000000000016213

**Published:** 2019-07-05

**Authors:** Jingyan Song, Zhengao Sun

**Affiliations:** a Shandong University of Traditional Chinese Medicine; b Reproductive and Genetic Center of Integrated Traditional and Western Medicine, The Affiliated Hospital of Shandong University of Traditional Chinese Medicine, Jinan, China.

**Keywords:** double trigger, empty follicle syndrome, luteinizing hormone threshold

## Abstract

**Rationale:**

The borderline form of empty follicle syndrome (EFS) is a phenomenon where only a few mature or immature oocytes are retrieved despite adequate response to controlled ovarian hyperstimulation (COH). It is a rare phenomenon with an unclear underlying mechanism, and there is currently no effective treatment.

**Patient concerns:**

The patient received 3 assisted reproductive technology cycles, and although her follicular development and estrogen levels were normal during COH, the outcome with respect to the oocytes obtained was unsatisfactory.

**Diagnoses:**

Borderline form of EFS.

**Interventions:**

In the context of undergoing GnRH-antagonist protocol, we implemented a double-trigger with human chorionic gonadotropin (hCG) after 6 hours of gonadotropin-releasing hormone agonist (GnRH-a) administration.

**Outcomes:**

Eleven oocytes were obtained (M I × 3, M II × 8), which underwent in vitro fertilization (IVF). After 18 hours, 7 oocytes showed normal fertilization, with 2 embryos formed 72 hours later (embryo rating, 6C II × 1, 9C II × 1); the embryos were then frozen.

**Lessons:**

Oocyte maturation and ovulation are time-dependent processes, and that different patients require different lengths/intervals of time for treatment. Therefore, the borderline form of EFS, in general, may be treatable, and our novel trigger method provides a new treatment option for such patients in the future.

## 1 Introduction

Coulam^[1]^ first discovered empty follicle syndrome (EFS) in 1986, and its definition was gradually modified. The definition of EFS today is a phenomenon where no oocytes are retrieved in a controlled ovarian hyperstimulation (COH) cycle even with multiple aspirations and flushings, despite normal increases in follicular development and estrogen levels and a normal gonadotropin beta subunit (β-hCG) level.^[2]^ Reports in the literature show that the typical incidence of EFS is between 0.045% and 3.4%.^[3]^ The occurrence of a borderline form of EFS was initially thought to be due to a human chorionic gonadotropin (hCG) injection error.^[4–6]^ Nikolettos^[7]^ found that oocyte retrieval was still not optimal even when the ovarian response was good and 10,000 U of hCG was injected (no oocytes were obtained in 1 cycle, and 2 oocytes were obtained from the other cycle; 1 oocyte was in MI and 1 degenerated). Nikolettos^[7]^ suggested that the EFS diagnosis was not rigorously defined, and therefore the definition of a borderline form of EFS was proposed to reflect this phenomenon.

The borderline form of EFS refers to a phenomenon where only a small number of mature or immature oocytes are obtained during a COH cycle despite good ovarian response (i.e., follicular development synchronized with estrogen growth) and injection of hCG.^[3,7]^ Although the difference between the borderline form of EFS and general EFS is vague and has no unified standard, the borderline form of EFS is more reflective of the dynamic variability of such phenomena relative to EFS. This suggests that EFS is not a permanent pathophysiologic state, rather that it can be treated with appropriate means. Therefore, after reviewing the results of our patient's 3 treatment cycles, we concluded that she can be temporarily diagnosed as manifesting a borderline form of EFS.

## 2 Case report

The patient was a 32-year-old woman with a history of infertility for 7 years. Her BMI was 22.22 kg/m^2^, and she showed irregular menstrual cycles with intervals of 30 to 90 days. In the past, she had received 6 cycles of drug-induced ovulation (clomiphene or urinary gonadotropins), which resulted in the growth of dominant follicles but no conceptions. Physical examination showed no obvious acne or hirsutism. An ultrasonogram showed ovarian polycystic changes, and hysterosalpingography (HSG) suggested bilateral fallopian tube insufficiency due to obstruction. Laboratory examination depicted the following hormonal concentrations in serum: basic follicle stimulation hormone (FSH), 4.46 U/L; LH, 11.61 U/L; estradiol (E_2_), 86 pg/mL; PRL, 23.6 ng/mL; and T, 0.25 ng/mL. Peripheral chromosomal examination displayed a 46, XX karyotype. The patient's husband was 38 years old and did not smoke or drink alcohol. The 5th WHO semen parameter standard was used to evaluate his semen and the results were within normal ranges. Chromosomal examination of the husband showed a 46, XY karyotype. The initial diagnosis was primary infertility, polycystic ovary syndrome (PCOS), and bilateral fallopian tube obstruction. The patient met the indications for assisted reproductive technology (ART), and in vitro fertilization (IVF) technology was used to assist in conception.

### 2.1 June 2015, first treatment cycle

In June 2015, the patient received a first cycle of IVF, with a gonadotropin-releasing hormone agonist (GnRH-a) long regimen initiated in the luteal phase. GnRH-a (Diphereline, 0.1 mg, France, Epson) administration commenced from the 20th day of the last menstrual period to downregulate pituitary function, and the treatment period lasted for 18 days. Serum sex hormones were evaluated on the first day of GnRH-a administration (day 5 of the menstrual cycle), and values were FSH, 3.11 U/L; LH, 1.17 U/L; and E_2_, 39 pg/mL. We then administered a combination therapy of r-FSH (Gonal-F 75 U, Merck, Germany) and HMG (75 U, Livzon Pharmaceutical), and the totals amounts of Gonal-F and HMG were 825 units each. The E_2_ levels were 346, 1150, and 3080 pg/mL on days 6, 9, and 12, respectively. On day 13 we observed the following hormone concentrations: E_2_, 4029 pg/mL; LH, 0.51 U/L; and PRL, 1.04 ng/mL; and bilateral ovaries ≥14 mm were observed. We noted 17 follicles (left ovarian diameter ≥ 17 mm, with 5 dominant follicles; right ovarian diameter ≥ 17 mm, with 4 dominant follicles). A dual trigger of 2000 U of hCG (Livzon Pharmaceutical) and 250 μg of r-hCG (Ovidrel, European Serono, France) was then provided. Ultrasound-guided vaginal puncture was performed to retrieve oocytes 36 hours later, but we could not retrieve oocytes from the left ovary even after repeated washings. Serum hormone concentrations were β-HCG, 64.27 U/L; E_2,_ 3209 pg/mL; PRL, 7.86 ng/mL; and LH, 0.78 U/L. After 2 hours, a more-experienced physician attempted to aspirate oocytes from the right ovary, but was unsuccessful. This cycle was then cancelled.

### 2.2 November 2015 to March 2016, second treatment cycle

In November 2015, a second cycle of IVF was begun, and we used a fixed GnRH antagonist protocol. From the first to fifth days (menstrual period days 3–8), we administered 5 mg/d of oral letrozole and 150 U of urinary gonadotropin (HMG). Gonadotropin-releasing hormone antagonist (GnRH-ant) (Cetrotide, 0.25 mg, Baxter, Germany) was administered from the 6th day to the trigger day. The treatment period lasted for 13 days, and a total of 2025 U of Exogenous gonadotropins were used. The serum E_2_ levels on days 1, 9, and 12 were 55, 483, and 2133 pg/mL, respectively. On day 13, we observed the following hormone concentrations in serum: E_2_, 2409 pg/mL; LH, 4.81 U/L; and PRL, 1.85 ng/mL; and observed bilateral ovaries ≥ 14 mm in diameter. We observed a total of 22 follicles (left ovarian diameter ≥ 17 mm, with 5 dominant follicles; right ovarian diameter ≥ 17 mm, with 8 dominant follicles). On day 13, 10,000 U of hCG and 250 μg of Ovidrel were injected. After 36 hours, 1 mature oocyte and 1 suspected oocyte (the latter appearing as a granulosa cumulus cell cluster that was later confirmed to be an immature oocyte) were obtained from the right ovary. The serum β-hCG level was 236.63 mIU/mL, E_2_ was 1456 pg/mL, and PRL was 13.13 ng/mL. After 2 hours, another puncture was performed and on oocyte was aspirated from the left ovary; diphereline (0.2 mg) was then injected despite the high bioavailability of serum hCG. However, there was still no retrievable oocyte from the left ovary 24 hours later. Spermatozoa provided normal fertilization after 18 hours of in vitro culture, and 2 grade-III embryos developed after 72 hours. In March 2016, although the replacement cycle frozen-thawed embryo transfer was itself replaced and 2 embryos were transplanted, the patient did not conceive.

### 2.3 November 2016, third treatment cycle

In November 2016, the patient received a third cycle of IVF. We still used the fixed GnRH-antagonist protocol, and 225 U of HMG was injected on the 14th day of the menstrual cycle. GnRH-ant (Cetrotide, 0.25 mg) was then administered from day 6 to the trigger day. The treatment period lasted for 11 days and a total of 2850 U of Exogenous gonadotropins were used. Serum E2 levels on days 1, 6, and 9 were 86, 1229, and 4814 pg/ml, respectively. On day 11 we detected in serum the following hormonal concentrations: E_2_, 5060 pg/mL; LH, 2.60 U/L; and PRL, 0.59 ng/mL; and observed bilateral ovaries ≥ 14 mm. We observed 12 follicles (left ovarian diameter ≥ 17 mm, with 2 dominant follicles; right ovarian diameter ≥ 17 mm, with 1 dominant follicle). We injected the first trigger of 0.2 mg of GnRHa at 3:00 p.m., and gave the second trigger of 250 μg of Ovidrel and 2000 U of hCG at 9:00 p.m. Thirty-six hours after the second trigger, ultrasound-guided vaginal puncture was performed and 11 oocytes were retrieved from the 2 ovaries (M I × 3, M II × 8). These oocytes were inseminated by IVF, and 7 fertilized normally after 18 hours. Two embryos developed 72 hours later (embryo rating, 6C II × 1, 9C II × 1), and the whole embryos were frozen.

## 3 Discussion

The patient received 3 ART cycles, and although her follicular development and estrogen levels were normal during COH, the outcome with respect to the oocytes obtained was atypical (see Table 1 for details).

**Table 1:**
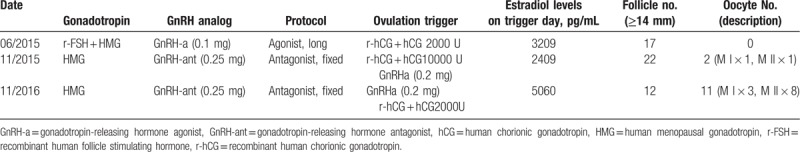
ART cycle description.

The success of oocyte retrieval is affected by 2 important factors: one is the dosage of the trigger, and the other is the time interval from trigger injection to oocyte retrieval. Our PCOS patient received 2000 U of hCG and 250 μg of Ovidrel in the first cycle, but the left ovary failed to produce oocytes; and we obtained no oocytes from the right ovary even after delaying the procedure for 2 hours. Then, in the second cycle, we administered 10,000 U of hCG and 250 μg of Ovidrel, and 2 oocytes were obtained. This indicated that the failure of the first cycle was due to inadequate dosage of the trigger medication and the short procedural delay. The oocyte was thereby presumably not fully exposed to the action of hCG and could not respond in a timely manner, such that the it failed to mature and ovulate. Some scholars have tried to predict the occurrence of the borderline form of EFS using a threshold value of serum β-HCG: Ndukwe^[8]^ defined it at 10 U/L, and Stevenson and Lashen^[2]^ at 40 U/L. In the first cycle, the patient's β-hCG level on the day of oocyte retrieval was 64.27 U/L, which was substantially higher than either of the aforementioned critical values. Therefore, hCG may not have been replenished appropriately. In the second cycle, although the trigger dose was increased to prevent the reduction in medication bioavailability, after 36 hours we only obtained 2 oocytes from the dominant follicles. We therefore injected 0.2 mg of diphereline after 2 hours, and the hCG exposure time was prolonged. After 24 hours, the remaining dominant follicles were punctured again, but no oocytes were obtained. The success of oocyte retrieval in the second cycle may be attributed to more attention given to this patient and the surgical skill of the physician. Abdalla^[9]^ found that the success rate of oocyte retrieval with 2,000 U of hCG was significantly lower than with either 5,000 U or 10,000 U of hCG. We speculate that the threshold for achieving a LH peak required for oocyte maturation differs between patients. It is certainly possible that the occurrence of the borderline form of EFS in our case was due to hCG dosage that did not achieve the minimal standard for oocyte maturation (i.e., less than that in a normal LH peak), and this resulted in a low oocyte retrieval rate.

Considering the above 2 important influencing factors, we improved the triggering strategy in the third cycle. Simultaneous to our extending the time from injection of trigger medication to oocyte retrieval, we also used a double-trigger method, with successful results. The present case suggests that oocyte maturation and ovulation are time-dependent processes, and that different patients require different lengths/intervals of time for treatment. Therefore, we speculate that some patients may take longer to complete cumulus expansion and ovulation, which would be crucial to oocyte retrieval. Therefore, the present occurrence of a borderline form of EFS was related to the inappropriate interval of 36 hours after the conventional physiologic trigger. In the present report, the double-trigger with GnRHa and hCG was effective on this borderline form of EFS. This indicates that the borderline form of EFS or EFS in general may be treatable, and provides a new treatment option for such patients in the future, which is promising for both patients and physicians.

## Author contributions

**Conceptualization:** Jingyan Song.

**Formal analysis:** Zhengao Sun.

**Resources:** Zhengao Sun.

**Validation:** Zhengao Sun.

**Writing – original draft:** Jingyan Song.

**Writing – review and editing:** Jingyan Song.
